# Editorial: Longitudinal data analysis in child and adolescent mental health

**DOI:** 10.3389/fpsyt.2022.1038190

**Published:** 2022-10-03

**Authors:** Tomoya Hirota, Eoin McElroy, Takeo Fujiwara, Joseph Boden

**Affiliations:** ^1^Department of Psychiatry and Behavioral Sciences, University of California, San Francisco, San Francisco, CA, United States; ^2^School of Psychology, Ulster University, Coleraine, United Kingdom; ^3^Department of Global Health Promotion, Tokyo Medical and Dental University, Tokyo, Japan; ^4^Department of Psychological Medicine, University of Otago, Christchurch, New Zealand

**Keywords:** child and adolescence psychiatry, child and adolescent mental health, longitudinal data analysis, longitudinal studies, psychopathology

Compared to adults, children and adolescents undergo rapid and dynamic changes in social, emotional, behavioral, and cognitive development and functioning (1, 2). As such, studies that track continuities and discontinuities of individual development, and that elucidate factors influencing different patterns of individual development, are essential in child and adolescent mental health research. Critical questions in this area include: which problems do children continue to exhibit over time; are there measurable characteristics specific to those children; what factors predict future psychopathologies; and what are the effects of early interventions and treatment on mental health outcomes? These questions are also important in clinical medicine, given that many psychiatric disorders and problems, including depression, anxiety, attention deficit hyperactivity disorder, and behavioral problems, can have their onset in childhood and persist into adolescence and adulthood (3). However, cross-sectional designs, frequently used in research for the simplicity of the data acquisition and the practicality in terms of cost and time, cannot provide answers to research questions focused on functioning over the life course.

In contrast, longitudinal study designs that follow the same individuals over multiple waves of data collection can shed light on complex developmental processes (4), providing evidence that can address questions such as those posed above. Additionally, with the development of standardized and valid assessment tools suitable for repeated measurements, and advances in statistical modeling and analysis, longitudinal research has considerable power to elucidate causal mechanisms of mental health disorders and other disorders. A single-cohort design, where a sample of a defined age is recruited at one point in time and followed up at subsequent intervals, is the most commonly used longitudinal design. A longitudinal sequential design, where two or more cohorts of differing ages are selected at the start of the study and are followed forwards, is another strategy that overcomes limitations in the single cohort study design, such as the inability to delineate age effects (developmental changes associated with age), period effects (variations in the time periods that affect all population regardless of age and cohort at the same time: war, infectious disease outbreaks, for example) and cohort effects (characteristics unique to the birth year of the cohort). To reflect this growing research area, we developed this Research Topic to present papers using longitudinal data analyses in child and adolescent mental health.

Several articles published in this collection provided findings related to predictive factors for future mental health problems through their longitudinal study designs. In a Korean community-based longitudinal study, 1,760 seventh-grade adolescents in Korea were followed for 2 years to examine the effects of exposure to online games before entering elementary school on Internet Gaming Disorder (IGD) occurrence during the secondary school years (Jeong et al.). The authors employed generalized-estimating-equation model and identified exposures to online games during preschool years as predictors for the high risk of IGD during the two-year study follow-up period (adjusted relative risk:1.69; 95%confidence interval:1.08–2.66). In another study conducted in the Netherland, the authors utilized longitudinal data prospectively collected from 2,523 individuals between 13 and 26 years of age in the Dutch Tracking Adolescents' Individual Lives Survey (Melo et al.). In this study, they examined whether reward sensitivity measured at age 13 predicted the course of multiple psychopathology domains over five measurement waves during the 14-year follow-up period, and found that reward sensitivity had a stable main effect on some psychopathology domains (anxiety, aggression), but that its effect increased over time on some domains (alcohol and cannabis use), indicating the transdiagnostic role of reward sensitivity in the course of the development of psychopathology between adolescence and adulthood. In addition to these two articles, Japanese researchers reported the positive influence of praise for the child's prosocial behaviors at 10 years of age on the child's depressive symptoms at age 12, and the impact of maternal stress related to child rearing during the first 3 years following birth on the child's ADHD symptoms at 12 years of age using data from the Tokyo Teen Cohort study, a population-based prospective study targeting adolescents (Nagaoka et al., Endo et al.). Adequate sample sizes in this cohort study (3,171 households for the first wave of data collection) allowed the researchers to identify statistically significant associations between the variables tested. In the second study from this research group, the researchers utilized data from the Maternal and Child Health Handbook, a home health book distributed by Japanese municipalities to all pregnant women who are expected to prospectively record their pregnancy and delivery and monitor the child's development, minimizing recall bias related to child-rearing stress.

In two articles, researchers employed cross-lagged analysis, an analytical strategy used to describe reciprocal relationships or directional influences between variables over time, to examine the association between family functioning and adolescents' depressive symptoms in China (Wang et al.), and between maternal internalizing problems (anxiety and depression) and the child's tics in early adolescence in Japan (Yagi et al.). In the first article, 1,301 Chinese middle school students underwent assessment for family functioning and depressive symptoms three times over 3 years. This study revealed the negative influence of the family function in the 7th grade on the child's depressive symptoms in the 8th grade, while the child's depressive symptoms in the 8th grade negatively impacted the family function a year after, suggesting a circular effect between family function and adolescent depressive symptoms. In the other article using data from the above-mentioned cohort study established in Tokyo, researchers identified bidirectional relationships between maternal depressive and anxious symptoms and the frequency of adolescents' tics, measured at ages 10 and 12. Findings from these two articles underscore the importance of advancing family-centered care in this field.

Longitudinal research also enables researchers to distinguish different trajectory patterns of the child's development. The researchers from Japan using the birth cohort data in Hamamatsu city employed parallel process multigroup latent class growth analysis to identify distinct trajectory patterns of three different domains of adaptive behaviors (communication, daily living skills, and socialization) and assessed sex differences in the trajectory structures and neurodevelopmental traits of children assigned to each trajectory class (Nishimura et al.). In this study, researchers identified four distinct trajectories and sex differences in the scale scores of adaptive behaviors and neurodevelopmental traits. In another study, researchers retrospectively collected data on healthcare utilization through medical chart review in children with neurodevelopmental disorders who presented to registered medical facilities in Japan (Aoki et al.). Data related to the number of outpatient consultations, history of hospitalization, and the use of multi-agency liaison over the last 5 years, repeatedly measured once every 6 months (i.e., 10-time points) were analyzed as longitudinal variables using cluster analysis. Findings revealed that while many children required consultations for a brief period, some needed continued support/consultations. In school-age children and adolescents, ADHD and other psychiatric comorbidities were associated with clusters indicating the continuous, longer use of healthcare.

This collection of articles demonstrates the scope and power of longitudinal research, and we hope that the findings are used to drive real change across a range of policy areas and intervention approaches. The selection of articles represents our collective belief that longitudinal research plays a vital role in understanding the social, environmental and family factors in early life that can affect health and wellbeing over the life course.

## Author contributions

TH drafted the editorial. All authors reviewed and commented on the draft and contributed to the final version of the editorial.

## Conflict of interest

The authors declare that the research was conducted in the absence of any commercial or financial relationships that could be construed as a potential conflict of interest.

## Publisher's note

All claims expressed in this article are solely those of the authors and do not necessarily represent those of their affiliated organizations, or those of the publisher, the editors and the reviewers. Any product that may be evaluated in this article, or claim that may be made by its manufacturer, is not guaranteed or endorsed by the publisher.
